# 2′-Methyl­pyrazolo[4′,3′:16,17]androst-5-en-3β-ol

**DOI:** 10.1107/S1600536809019539

**Published:** 2009-05-29

**Authors:** Hu-ling Zheng, Peng Xia, Ying Chen

**Affiliations:** a Department of Medicinal Chemistry, School of Pharmacy, Fudan University, 138 Yixueyuan Road Shanghai 200032, People’s Republic of China

## Abstract

In the title compound, C_21_H_30_N_2_O, there are five fused rings. The *A* and *C* rings adopt chair conformations, ring *B* adopts an 8β,9α-half-chair conformation and ring *D* adopts a 14α-envelope conformation. The pyrazole ring is planar. Inter­molecular O—H⋯N hydrogen bonds [H⋯N = 1.88 (5) Å] help to stabilize the crystal structure. The absolute structure was deduced from those of the starting materials.

## Related literature

For general background, see: Kashiwada *et al.* (1996 ▶); Spek (2009 ▶).
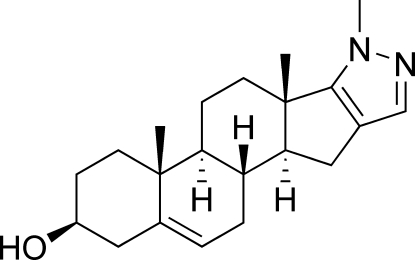


## Experimental

### 

#### Crystal data

C_21_H_30_N_2_O
*M* *_r_* = 326.47
Orthorhombic, 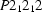

*a* = 11.779 (4) Å
*b* = 27.996 (10) Å
*c* = 6.361 (2) Å
*V* = 2097.6 (12) Å^3^
*Z* = 4
Mo *K*α radiation
μ = 0.06 mm^−1^
*T* = 293 K
0.20 × 0.10 × 0.08 mm

#### Data collection

Bruker SMART CCD area-detector diffractometer
Absorption correction: multi-scan (*SADABS*; Sheldrick, 1996 ▶) *T*_min_ = 0.988, *T*_max_ = 0.995
10038 measured reflections
2633 independent reflections
1670 reflections with *I* > 2σ(*I*)
*R*_int_ = 0.089

#### Refinement

*R*[*F* ^2^ > 2σ(*F* ^2^)] = 0.065
*wR*(*F* ^2^) = 0.178
*S* = 0.99
2633 reflections
224 parameters
H atoms treated by a mixture of independent and constrained refinement
Δρ_max_ = 0.26 e Å^−3^
Δρ_min_ = −0.15 e Å^−3^

### 

Data collection: *SMART* (Bruker, 2000 ▶); cell refinement: *SAINT* (Bruker, 2000 ▶); data reduction: *SAINT*; program(s) used to solve structure: *SHELXS97* (Sheldrick, 2008 ▶); program(s) used to refine structure: *SHELXL97* (Sheldrick, 2008 ▶); molecular graphics: *SHELXTL* (Sheldrick, 2008 ▶); software used to prepare material for publication: *SHELXTL*.

## Supplementary Material

Crystal structure: contains datablocks global, I. DOI: 10.1107/S1600536809019539/rk2148sup1.cif

Structure factors: contains datablocks I. DOI: 10.1107/S1600536809019539/rk2148Isup2.hkl

Additional supplementary materials:  crystallographic information; 3D view; checkCIF report

## Figures and Tables

**Table 1:** Hydrogen-bond geometry (Å, °)

*D*—H⋯*A*	*D*—H	H⋯*A*	*D*⋯*A*	*D*—H⋯*A*
O1—H1⋯N2^i^	0.96 (5)	1.88 (6)	2.813 (5)	163 (5)

Symmetry code: (i) 
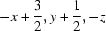
.

**Fig. 1.**
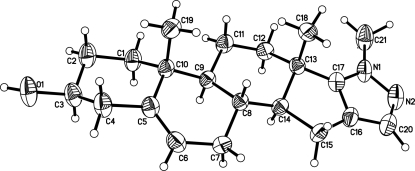
The molecular structure of the title compound with the atom numbering scheme. The displacement ellipsoids are drawn at the 50% probability level. The H atoms are presented as a small spheres of arbitrary radius.

## supplementary crystallographic information

### Comment

The 3-*O*-(2',2'-dimethylsuccinyl)-betulinic acid, a derivative of natural product betulinic acid, was identified as a potent anti-*HIV* (human immunodificiency virus) agent with remarkable active value (Kashiwada *et al.*, 1996). Based on the structure and bioactivity of 3-*O*-(2',2'-dimethylsuccinyl)-betulinic acid, we tried to synthesize some of its steroidal analogs with a heterocycle fused *E* ring. During synthesizing a target compound 3β-*O*-(2'',2''-dimethylsuccinyl)-4,4-dimethyl-androst-[17,16- c]-(2'-methyl)pyrazole, an important intermediate, 3β-hydroxy-androst-5-en-[17,16-c]-(2'-methyl)pyrazole, was obtained and its molecular structure was reported here.

Fig.1 shows the molecular structure of the title compound. This compound is a five-ring-fused compound. Ring *A* and ring *C* adopt chair conformations in each molecule. The C5–C6 distance of 1.340 (5) Å conform the localization of a double bond at this position. As a result of this double bond, the geometry around C5 is planar and hence ring *B* adopt 8β,9α-half-chair conformation. The ring *D* assumes 14α-envelope conformation. The pyrazole *E* ring is essentially planar. Intermolecular O1–H1···N2^i^ hydrogen bond with parameters O1–H1 = 0.96 (5)Å, H1···N2^i^ = 1.88 (6)Å, O1···N2^i^ = 2.813 (5)Å and angle O1–H1···N2^i^ = 163 (5)° (symmetry code: (i) -*x*+3/2, *y*+1/2, -*z*) help to stablize the crystal structure.

### Experimental

3β-Hydroxy-16-hydroxymethylene-androst-5-en-17-one (500 mg, 1.58 mmol) was dissolved in 10 ml EtOH, and methylhydrazine (120 mg, 2.61 mmol) was added. The resulting mixture was stirred for 2 h at room temperature, and 100 ml H_2_O was added. After filtered, washed with water and dried, crude product of title compound (520 mg) was got. The crude product was purified by chromatography with petroleum ether/ EtOAc (10:3) as eluent and recrystallized from tetrahydrofuran to obtain its single-crystal for X-ray diffraction analysis.

### Refinement

All H atoms except H1 were positioned geometrically and refined using a riding model with C–H = 0.93Å for aromatic H atoms and C–H = 0.96Å for methyl H atoms, and refine in riding mode with *U*_iso_(H) = 1.2*U*_eq_(C) for aromatic H atoms and *U*_iso_(H) = 1.5*U*_eq_(C) for methyl H atoms. H1 had been found on the different Fourier map and refined without bond restrain. In the absence of significant anomalous scattering, 845 Friedel pairs were merged and all Δf" values to be set to zero.

### Crystal data

**Table:**

C_21_H_30_N_2_O	*F*(000) = 712
*M**_r_* = 326.47	*D*_x_ = 1.034 Mg m^−^^3^
Orthorhombic, *P*2_1_2_1_2	Mo *K*α radiation, λ = 0.71073 Å
Hall symbol: P 2 2ab	Cell parameters from 985 reflections
*a* = 11.779 (4) Å	θ = 2.3–21.3°
*b* = 27.996 (10) Å	µ = 0.06 mm^−^^1^
*c* = 6.361 (2) Å	*T* = 293 K
*V* = 2097.6 (12) Å^3^	Column, colourless
*Z* = 4	0.20 × 0.10 × 0.08 mm

### Data collection

**Table:**

Bruker SMART CCD area-detector diffractometer	2633 independent reflections
Radiation source: fine-focus sealed tube	1670 reflections with *I* > 2σ(*I*)
graphite	*R*_int_ = 0.089
φ and ω scans	θ_max_ = 27.1°, θ_min_ = 1.5°
Absorption correction: multi-scan (*SADABS*; Sheldrick, 1996)	*h* = −11→15
*T*_min_ = 0.988, *T*_max_ = 0.995	*k* = −35→27
10038 measured reflections	*l* = −8→8

### Refinement

**Table:**

Refinement on *F*^2^	Secondary atom site location: difference Fourier map
Least-squares matrix: full	Hydrogen site location: inferred from neighbouring sites
*R*[*F*^2^ > 2σ(*F*^2^)] = 0.065	H atoms treated by a mixture of independent and constrained refinement
*wR*(*F*^2^) = 0.178	*w* = 1/[σ^2^(*F*_o_^2^) + (0.097*P*)^2^] where *P* = (*F*_o_^2^ + 2*F*_c_^2^)/3
*S* = 0.99	(Δ/σ)_max_ < 0.001
2633 reflections	Δρ_max_ = 0.26 e Å^−^^3^
224 parameters	Δρ_min_ = −0.15 e Å^−^^3^
0 restraints	Absolute structure: Flack (1983)
Primary atom site location: structure-invariant direct methods	Flack parameter: 0 (10)

### Special details

**Table:**

Experimental. Compound contains disordered unassigned solvent (tetrahydrofuran), which was SQEEZED with program *PLATON* (Spek, 2003). Solvent is not contained in chemical formula and quantities derived thereof. Informations on the SQUEEZE procedure are given subsequently.
Geometry. All s.u.'s (except the s.u. in the dihedral angle between two l.s. planes) are estimated using the full covariance matrix. The cell s.u.'s are taken into account individually in the estimation of s.u.'s in distances, angles and torsion angles; correlations between s.u.'s in cell parameters are only used when they are defined by crystal symmetry. An approximate (isotropic) treatment of cell s.u.'s is used for estimating s.u.'s involving l.s. planes.
Refinement. Refinement of *F*^2^ against ALL reflections. The weighted *R*-factor *wR* and goodness of fit *S* are based on *F*^2^, conventional *R*-factors *R* are based on *F*, with *F* set to zero for negative *F*^2^. The threshold expression of *F*^2^ > σ(*F*^2^) is used only for calculating *R*-factors(gt) *etc*. and is not relevant to the choice of reflections for refinement. *R*-factors based on *F*^2^ are statistically about twice as large as those based on *F*, and *R*-factors based on ALL data will be even larger.

### Fractional atomic coordinates and isotropic or equivalent isotropic displacement parameters (Å^2^)

**Table:**

	*x*	*y*	*z*	*U*_iso_*/*U*_eq_	
O1	0.8092 (3)	0.49975 (10)	0.3404 (5)	0.0957 (11)	
H1	0.809 (4)	0.5288 (17)	0.261 (10)	0.114 (16)*	
N1	0.7846 (3)	0.11087 (10)	−0.1171 (5)	0.0720 (9)	
N2	0.7158 (4)	0.07461 (12)	−0.0492 (6)	0.0859 (11)	
C1	0.8428 (4)	0.38834 (12)	0.0088 (6)	0.0682 (10)	
H1A	0.8983	0.3797	−0.0973	0.082*	
H1B	0.7686	0.3884	−0.0573	0.082*	
C2	0.8696 (4)	0.43932 (13)	0.0914 (7)	0.0774 (12)	
H2A	0.9455	0.4400	0.1503	0.093*	
H2B	0.8667	0.4619	−0.0241	0.093*	
C3	0.7851 (4)	0.45347 (13)	0.2566 (7)	0.0740 (11)	
H3	0.7090	0.4537	0.1945	0.089*	
C4	0.7871 (4)	0.41834 (13)	0.4390 (7)	0.0746 (11)	
H4A	0.8602	0.4200	0.5089	0.090*	
H4B	0.7290	0.4271	0.5401	0.090*	
C5	0.7665 (3)	0.36782 (12)	0.3634 (6)	0.0583 (9)	
C6	0.6856 (3)	0.34023 (13)	0.4488 (6)	0.0674 (10)	
H6	0.6389	0.3540	0.5500	0.081*	
C7	0.6648 (3)	0.28952 (13)	0.3943 (6)	0.0613 (9)	
H7A	0.5958	0.2873	0.3118	0.074*	
H7B	0.6536	0.2714	0.5225	0.074*	
C8	0.7623 (3)	0.26762 (10)	0.2707 (5)	0.0482 (8)	
H8	0.8270	0.2627	0.3650	0.058*	
C9	0.7973 (3)	0.30251 (11)	0.0934 (5)	0.0496 (8)	
H9	0.7269	0.3110	0.0204	0.060*	
C10	0.8443 (3)	0.35012 (11)	0.1862 (5)	0.0513 (8)	
C11	0.8757 (3)	0.28075 (12)	−0.0755 (6)	0.0610 (9)	
H11A	0.8766	0.3019	−0.1963	0.073*	
H11B	0.9523	0.2795	−0.0199	0.073*	
C12	0.8425 (3)	0.23078 (12)	−0.1496 (5)	0.0578 (9)	
H12A	0.9017	0.2181	−0.2396	0.069*	
H12B	0.7730	0.2325	−0.2309	0.069*	
C13	0.8252 (3)	0.19741 (12)	0.0396 (5)	0.0508 (8)	
C14	0.7296 (3)	0.22009 (12)	0.1721 (5)	0.0497 (8)	
H14	0.6698	0.2279	0.0707	0.060*	
C15	0.6785 (3)	0.17936 (12)	0.3103 (6)	0.0609 (10)	
H15A	0.7204	0.1751	0.4403	0.073*	
H15B	0.5989	0.1848	0.3415	0.073*	
C16	0.6951 (3)	0.13810 (12)	0.1623 (6)	0.0619 (9)	
C17	0.7721 (3)	0.14936 (12)	0.0079 (6)	0.0598 (9)	
C18	0.9367 (3)	0.19017 (13)	0.1615 (7)	0.0654 (10)	
H18A	0.9213	0.1735	0.2904	0.098*	
H18B	0.9698	0.2207	0.1925	0.098*	
H18C	0.9885	0.1718	0.0777	0.098*	
C19	0.9641 (3)	0.34398 (13)	0.2742 (7)	0.0667 (10)	
H19A	0.9652	0.3175	0.3703	0.100*	
H19B	0.9862	0.3726	0.3466	0.100*	
H19C	1.0160	0.3379	0.1609	0.100*	
C20	0.6632 (4)	0.09124 (14)	0.1184 (8)	0.0744 (11)	
H20	0.6113	0.0738	0.1975	0.089*	
C21	0.8604 (5)	0.10396 (15)	−0.2928 (8)	0.0969 (16)	
H21A	0.9134	0.1300	−0.2991	0.145*	
H21B	0.8173	0.1028	−0.4207	0.145*	
H21C	0.9010	0.0745	−0.2753	0.145*	

### Atomic displacement parameters (Å^2^)

**Table:**

	*U*^11^	*U*^22^	*U*^33^	*U*^12^	*U*^13^	*U*^23^
O1	0.149 (3)	0.0445 (15)	0.094 (2)	0.0112 (17)	−0.004 (2)	0.0059 (15)
N1	0.106 (2)	0.0424 (17)	0.067 (2)	0.0002 (17)	0.002 (2)	0.0005 (14)
N2	0.128 (3)	0.0485 (19)	0.081 (3)	−0.006 (2)	−0.010 (2)	0.0001 (17)
C1	0.094 (3)	0.053 (2)	0.058 (2)	−0.0022 (19)	0.001 (2)	0.0149 (17)
C2	0.117 (3)	0.051 (2)	0.065 (3)	0.000 (2)	0.002 (2)	0.0167 (18)
C3	0.103 (3)	0.049 (2)	0.070 (3)	0.014 (2)	0.002 (2)	0.0118 (18)
C4	0.107 (3)	0.051 (2)	0.066 (3)	0.007 (2)	0.007 (2)	0.0039 (18)
C5	0.077 (2)	0.052 (2)	0.0456 (19)	0.0034 (18)	0.0003 (18)	0.0079 (15)
C6	0.079 (2)	0.063 (2)	0.061 (2)	0.0153 (19)	0.0104 (19)	0.0022 (18)
C7	0.0549 (18)	0.062 (2)	0.067 (2)	−0.0033 (16)	0.0072 (17)	0.0038 (17)
C8	0.0528 (17)	0.0477 (18)	0.0443 (18)	0.0005 (13)	0.0003 (15)	0.0059 (13)
C9	0.0541 (17)	0.0499 (18)	0.0448 (19)	−0.0011 (14)	−0.0031 (14)	0.0082 (14)
C10	0.0623 (19)	0.0460 (18)	0.046 (2)	−0.0007 (15)	0.0033 (15)	0.0095 (14)
C11	0.075 (2)	0.059 (2)	0.049 (2)	−0.0049 (18)	0.0132 (17)	0.0069 (16)
C12	0.068 (2)	0.062 (2)	0.043 (2)	0.0023 (16)	0.0073 (17)	0.0037 (15)
C13	0.0562 (17)	0.0490 (19)	0.0472 (19)	0.0049 (15)	0.0012 (14)	0.0024 (14)
C14	0.0499 (17)	0.0526 (18)	0.0466 (19)	−0.0060 (15)	−0.0022 (14)	0.0063 (14)
C15	0.066 (2)	0.056 (2)	0.060 (2)	−0.0136 (16)	0.0087 (17)	0.0066 (17)
C16	0.068 (2)	0.047 (2)	0.071 (3)	−0.0079 (16)	−0.0052 (19)	0.0035 (17)
C17	0.070 (2)	0.052 (2)	0.057 (2)	−0.0006 (17)	−0.0049 (19)	0.0013 (16)
C18	0.0574 (19)	0.066 (2)	0.073 (3)	0.0055 (17)	−0.0036 (18)	0.008 (2)
C19	0.063 (2)	0.065 (2)	0.072 (3)	−0.0086 (18)	−0.0060 (19)	0.0068 (19)
C20	0.086 (3)	0.058 (2)	0.079 (3)	−0.013 (2)	−0.008 (2)	0.013 (2)
C21	0.159 (4)	0.061 (3)	0.071 (3)	0.004 (3)	0.025 (3)	−0.004 (2)

### Geometric parameters (Å, °)

**Table:**

O1—C3	1.429 (5)	C9—C10	1.559 (5)
O1—H1	0.96 (5)	C9—H9	0.9800
N1—C17	1.347 (5)	C10—C19	1.527 (5)
N1—N2	1.369 (5)	C11—C12	1.527 (5)
N1—C21	1.443 (6)	C11—H11A	0.9700
N2—C20	1.317 (6)	C11—H11B	0.9700
C1—C2	1.553 (5)	C12—C13	1.537 (5)
C1—C10	1.555 (4)	C12—H12A	0.9700
C1—H1A	0.9700	C12—H12B	0.9700
C1—H1B	0.9700	C13—C17	1.497 (5)
C2—C3	1.501 (6)	C13—C18	1.538 (5)
C2—H2A	0.9700	C13—C14	1.543 (4)
C2—H2B	0.9700	C14—C15	1.560 (4)
C3—C4	1.521 (6)	C14—H14	0.9800
C3—H3	0.9800	C15—C16	1.503 (5)
C4—C5	1.513 (5)	C15—H15A	0.9700
C4—H4A	0.9700	C15—H15B	0.9700
C4—H4B	0.9700	C16—C17	1.374 (5)
C5—C6	1.341 (5)	C16—C20	1.393 (5)
C5—C10	1.535 (5)	C18—H18A	0.9600
C6—C7	1.482 (5)	C18—H18B	0.9600
C6—H6	0.9300	C18—H18C	0.9600
C7—C8	1.520 (5)	C19—H19A	0.9600
C7—H7A	0.9700	C19—H19B	0.9600
C7—H7B	0.9700	C19—H19C	0.9600
C8—C14	1.521 (4)	C20—H20	0.9300
C8—C9	1.548 (4)	C21—H21A	0.9600
C8—H8	0.9800	C21—H21B	0.9600
C9—C11	1.542 (5)	C21—H21C	0.9600
			
C3—O1—H1	125 (3)	C1—C10—C9	108.0 (3)
C17—N1—N2	110.0 (3)	C12—C11—C9	115.1 (3)
C17—N1—C21	129.2 (3)	C12—C11—H11A	108.5
N2—N1—C21	120.7 (3)	C9—C11—H11A	108.5
C20—N2—N1	105.8 (3)	C12—C11—H11B	108.5
C2—C1—C10	112.6 (3)	C9—C11—H11B	108.5
C2—C1—H1A	109.1	H11A—C11—H11B	107.5
C10—C1—H1A	109.1	C11—C12—C13	110.4 (3)
C2—C1—H1B	109.1	C11—C12—H12A	109.6
C10—C1—H1B	109.1	C13—C12—H12A	109.6
H1A—C1—H1B	107.8	C11—C12—H12B	109.6
C3—C2—C1	110.2 (3)	C13—C12—H12B	109.6
C3—C2—H2A	109.6	H12A—C12—H12B	108.1
C1—C2—H2A	109.6	C17—C13—C12	119.7 (3)
C3—C2—H2B	109.6	C17—C13—C18	107.8 (3)
C1—C2—H2B	109.6	C12—C13—C18	111.2 (3)
H2A—C2—H2B	108.1	C17—C13—C14	97.9 (3)
O1—C3—C2	111.6 (3)	C12—C13—C14	105.9 (3)
O1—C3—C4	107.4 (4)	C18—C13—C14	113.7 (3)
C2—C3—C4	110.7 (3)	C8—C14—C13	113.6 (2)
O1—C3—H3	109.0	C8—C14—C15	120.3 (3)
C2—C3—H3	109.0	C13—C14—C15	106.8 (3)
C4—C3—H3	109.0	C8—C14—H14	104.9
C5—C4—C3	111.1 (3)	C13—C14—H14	104.9
C5—C4—H4A	109.4	C15—C14—H14	104.9
C3—C4—H4A	109.4	C16—C15—C14	99.1 (3)
C5—C4—H4B	109.4	C16—C15—H15A	111.9
C3—C4—H4B	109.4	C14—C15—H15A	111.9
H4A—C4—H4B	108.0	C16—C15—H15B	111.9
C6—C5—C4	121.6 (3)	C14—C15—H15B	111.9
C6—C5—C10	122.4 (3)	H15A—C15—H15B	109.6
C4—C5—C10	116.0 (3)	C17—C16—C20	104.5 (4)
C5—C6—C7	125.1 (3)	C17—C16—C15	110.9 (3)
C5—C6—H6	117.5	C20—C16—C15	144.6 (4)
C7—C6—H6	117.5	N1—C17—C16	108.1 (3)
C6—C7—C8	112.5 (3)	N1—C17—C13	138.8 (4)
C6—C7—H7A	109.1	C16—C17—C13	112.7 (3)
C8—C7—H7A	109.1	C13—C18—H18A	109.5
C6—C7—H7B	109.1	C13—C18—H18B	109.5
C8—C7—H7B	109.1	H18A—C18—H18B	109.5
H7A—C7—H7B	107.8	C13—C18—H18C	109.5
C7—C8—C14	112.0 (3)	H18A—C18—H18C	109.5
C7—C8—C9	108.9 (3)	H18B—C18—H18C	109.5
C14—C8—C9	108.6 (3)	C10—C19—H19A	109.5
C7—C8—H8	109.1	C10—C19—H19B	109.5
C14—C8—H8	109.1	H19A—C19—H19B	109.5
C9—C8—H8	109.1	C10—C19—H19C	109.5
C11—C9—C8	114.7 (3)	H19A—C19—H19C	109.5
C11—C9—C10	112.9 (3)	H19B—C19—H19C	109.5
C8—C9—C10	111.0 (3)	N2—C20—C16	111.6 (4)
C11—C9—H9	105.8	N2—C20—H20	124.2
C8—C9—H9	105.8	C16—C20—H20	124.2
C10—C9—H9	105.8	N1—C21—H21A	109.5
C19—C10—C5	108.6 (3)	N1—C21—H21B	109.5
C19—C10—C1	110.7 (3)	H21A—C21—H21B	109.5
C5—C10—C1	107.7 (3)	N1—C21—H21C	109.5
C19—C10—C9	111.7 (3)	H21A—C21—H21C	109.5
C5—C10—C9	110.0 (3)	H21B—C21—H21C	109.5

### Hydrogen-bond geometry (Å, °)

**Table:**

*D*—H···*A*	*D*—H	H···*A*	*D*···*A*	*D*—H···*A*
O1—H1···N2^i^	0.96 (5)	1.88 (6)	2.813 (5)	163 (5)

Symmetry codes: (i) −*x*+3/2, *y*+1/2, −*z*.
